# Cross-sectional survey of underreported violence experienced by adolescents: a study from Indonesia

**DOI:** 10.1186/s12889-021-12427-8

**Published:** 2022-01-08

**Authors:** Yoni Syukriani, A. Noviandhari, N. Arisanti, E. P. Setiawati, V. K. Rusmil, M. Dhamayanti, N. Sekarwana

**Affiliations:** 1 Department of Forensic and Legal Medicine, Faculty of Medicine, Universitas Padjadjaran, Jalan Raya Bandung-Sumedang KM 21, Jatinangor, Sumedang, Bandung, West Java 45363 Indonesia; 2 Department of Children Health, Faculty of Medicine, Universitas Padjadjaran, Bandung, Indonesia; 3 Department of Public Health, Faculty of Medicine, Universitas Padjadjaran, Bandung, Indonesia

**Keywords:** Adolescents, Physical violence, Verbal violence, Sexual violence, Risk factor, Medicolegal, Indonesia

## Abstract

**Background:**

Global studies on adolescent victims of violence require serious attention due to the possibility that underreported cases may be higher than official records indicate. Since Indonesia expects to witness a demographic bonus, extensive research is needed to strengthen early detection, case handling, and prevention. Here, we report the outcomes of a survey on physical, verbal, and sexual violence experienced by adolescents in West Java, an Indonesian province inhabited by 18% of the country’s total population.

**Methods:**

We conducted a cross-sectional survey in 2017 using the International Society for the Prevention of Child Abuse and Neglect (ISPCAN) Child Abuse Screening Tool for Children (ICAST-C) questionnaire for detecting child abuse; an expert panel translated, simplified, and validated it based on a theoretical framework that combines paediatrics, public health, and medicolegal perspectives. We aimed to cover a large sample size and explore three types of violence (physical, verbal, and sexual) that have high evidentiary value in the forensic context. The respondents were adolescents in the first and second grades of middle school (12 to 14 years old) and high school (15 to 17 years old) in seven cities/municipalities in the province, selected through several stages of simple random sampling (*N* = 3452). We analysed the samples through univariate (percentage), odds ratio (OR), comparison, correlation, and correspondence analyses.

**Results:**

The results showed that 78.7% of the adolescents experienced violence in 2017, comprising those who encountered at least one incidence of physical violence (43.1%), verbal violence (12.2%), and sexual violence (4.5%). Data overlap includes 14.3% who experienced one type of violence in 2017, 7.4% who experienced two forms of violence, and 1.4% who underwent all three kinds of violence. The offenders were mainly adolescents across all types of violence, except for being forced to engage in sexual intercourse. Several victims of sexual violence did not state who the offenders were. Further, several characteristics showed a higher chance of experiencing violence than other characteristics, especially for adolescents who were still in middle school and those who lived only with their mothers. Correspondence analysis suggested subtle differences between characteristics.

**Conclusion:**

We expect this study to help identify risk and protective factors that are essential to strengthening early detection efforts, decisive medicolegal examinations, case handling, and policy-making.

**Supplementary Information:**

The online version contains supplementary material available at 10.1186/s12889-021-12427-8.

## Introduction

Cases of violence against children and adolescents have been a serious problem worldwide and have caused physical injuries, sexual and reproductive problems, and mental disorders [1–4]. The concern is the serious consequences due to adolescents’ physical, sexual, mental development, and the low rate of reporting and difficulty in early detection. The most salient types of violence experienced by children and adolescents are school-related violence, bullying and fighting, sexual violence and abuse, corporal discipline, and lethal violence [5–7]. Offenders can be fellow teenagers (peers or dating partners) or adults who should have a protective role, such as school staff, parents, or relatives [7, 8]. The impacts are physical and hinder in mental development, consequently triggering a generational burden.

One of the biggest problems with violence against adolescents is that the number of report-based incidents is small, and of these, only a small proportion can be validated by law enforcement [9]. The low reporting rate may be related to the reluctance to report, limited knowledge, and different perspectives on violence [10, 11]. The data obtained are usually based on official reports from government ministries/agencies, cases reported to law enforcement agencies, patients admitted to hospitals, or data from non-government organisations. In the Indonesian context, data could come from the Ministry of Women’s Empowerment and Child Protection (MoWECP), the National Commission on Child Protection, the Integrated Service Centre for Women Empowerment and Children (P2TP2A), and the Indonesian Association of Paediatricians. The rate of violence among adolescents between countries is influenced by age range, the data collection approach, the parameters of violence used, and the statistical approach used. An ICAST questionnaire-based study in 2009 showed that violence exposure among children in Colombia, India, Russia, and Iceland was 51.8, 40.1, 60.4, and 25.9%, respectively [12], while adolescent victimisation in Mexico reached 44% [2]. A number based on a questionnaire survey is usually higher than what is reported in official data. Studies focused on violent (physical or psychological) discipline in low- and middle-income countries can reach up to almost 95% [13, 14], although some studies have indicated a declining trend in certain countries [14].

Interestingly, in the context of Asia and the Pacific, violence against adolescents is often associated more with disciplined approaches at home and school and with fighting and bullying [15–18]. Data from the police and judicial system have long been considered the most reliable; however, figures depend heavily on people being willing to report incidents. Several studies have pointed out a significant difference between police data and hospital emergency data [9, 10]. From a forensic angle, the low success rate of legal prosecution may be due to underreporting and weak case handling [19, 20]. In addition, the thesis on corporal violence can be one explanation for why forensic evidence and law enforcement are becoming increasingly difficult.

The statistics of violence against children and adolescents in Indonesia show inconsistent figures between government agencies and various local studies, including different definitions, because they are adjusted to the respective objectives of recording cases [21]. As the world’s fourth most populous country, 46.8 million (18%) of Indonesia’s inhabitants are aged 10 to 19 [22, 23]. In general, the crime rate has fallen in the past three years, from 129/100,000 people in 2017 to 103/100,000 in 2019. The official data on violence or crimes involving child victims are limited only to child employment (2114 cases in 2019), which has risen from 1832 in 2015. Children who were crime victims in 2015 accounted for 6.25% of victims, which increased to 6.86% in 2019 [24, 25], and rose from 3700/year to 4615 in 7 months during the COVID-19 pandemic [26].

As suggested by previous research, we think it is necessary to better understand the epidemiology, risk factors, prevalence, consequences, interventions, and forensic and medicolegal aspects [4, 27–29] related to violence experienced by adolescents. Our goal was to evaluate cases of underreported violence in adolescents; and to identify risk and protective factors that are essential for healthcare providers to engage in early case detection, decisive forensic examinations, and the development of preventive policies. We used a combination of a public health and forensic theoretical framework by evaluating the epidemiological aspects of violence experienced by adolescents (physical, verbal and sexual) that have the potential to be forensically proven, but which have not been reported to healthcare personnel or law enforcement for various reasons [21, 30, 31]. Understanding the evidentiary value of information will guide the detection, examination, and handling of individual cases [10], and will serve as a reference for prevention. Although we explored three types of violence, physical violence was the main focus of our analysis.

## Methods

### The respondents

We performed this quantitative study in 2017 by collecting cross-sectional data from adolescent respondents younger than 18 years old, whom we grouped by school level of the first and second grades of middle school (range = 12 to 14) and the first and second grades of high school (range = 15 to 17) in cities and municipalities of West Java. We excluded students from the third grade of middle school and high school due to their schedule for national exams. All protocols were in accordance with the national and international ethics guidelines for involving human subjects in research, and had passed ethical clearance before the respondents were involved. We gathered the data through several stages of simple random sampling. As a result, we selected three cities and four municipalities. According to 2016 data, they had a population of 12.7 million people (26.8% of West Java’s population), with 2.6 million inhabitants aged 10 to 19. Cities and municipalities are differentiated based on several characteristics. We performed two simple random samplings to select 16 middle schools and 16 high schools, and then identified 48 middle school classes and 48 high school classes. We drew 40 students from each class, representing grades 7 to 12. All research protocols for involving humans followed the guidelines of national and international norms on research ethics. After the research protocol had passed the review process from an institutional research ethics committee, we distributed informed consent forms to ask for permission from parents/guardians before adolescents could become respondents and fill out the questionnaire. The total number of completed questionnaires returned was 3452.

### Questionnaire

We used the International Society for the Prevention of Child Abuse and Neglect (ISPCAN) Child Abuse Screening Tool for Children (ICAST-C) questionnaire to detect violence experienced by children [12, 32]. An expert panel translated, selected, simplified and tested the questionnaire for validation; this was followed by reliability tests [33, 34]. The questions regarding subject characteristics are city category (living in a city/municipality), gender, school level (middle school or high school), ethnic group, religion, the respondent’s position in the family, number of siblings, home environment (living with both parents, only with the father, only with the mother, with foster parents, not in a family home), and parents’ education level. In addition, by considering the Indonesian Criminal Code, we selected questions on violence. The questions comprised 18 items physical violence, two items on verbal violence, and four questions on sexual violence experienced by the respondents in 2017 (the study year), as well as in the past. Each question about violence was followed by a question about the offender, whether the offender(s) was/were male, female, a child/children, an adult/adults, or a combination of these. Since the respondents filled out the questionnaire on paper, there may have been questions that they either missed or failed to answer.

### Statistical analyses

We analysed the data using univariate (percentage), odds ratio (OR), comparison, correlation, and correspondence analyses. We performed univariate analysis to show the profile of each respondent, while we conducted OR analysis to examine the possibility of several characteristics being risk and protective factors, namely the city category, school level, gender, the respondent’s position in the family, number of siblings, type of home where the respondent was living, and parents’ education level. With the comparison test, we aimed to detect significant differences between the respondents’ characteristics and various types of violence experienced, while the purpose of the correlation test was to look for possible characteristics that might play a role in experiences of violence. With multivariate analysis, we intended to reveal subtle differences between the characteristics. For univariate analysis and OR, we grouped the incidences of violence experienced by the respondents into 23 attributes based on the types of violence and the timeframe (see Table 1):

**Table 1:** Attributes used to analyse experiences of violence

No.	Attribute
1.	At least one type of violence this year^a^
2.	At least one type of violence in the past^b^
3.	Any type of violence, this year^a^ and in the past^b^
4.	At least one form of physical violence this year^a^
5.	At least one form of physical violence in the past^b^
6.	Any physical violence, this year^a^ and in the past^b^
7.	At least one form of verbal violence this year^a^
8.	At least one form of verbal violence in the past^b^
9.	Any verbal violence, this year^a^ and in the past
10.	At least one form of sexual violence this year^a^
11.	At least one form of sexual violence in the past^b^
12.	Any sexual violence, this year^a^ and in the past^b^
13.	One form of physical violence only
14.	One form of verbal violence only
15.	One form of sexual violence only
16.	More than one form of physical violence
17.	More than one form of verbal violence
18.	More than one form of sexual violence
19.	Combination of physical and verbal violence
20.	Combination of physical and sexual violence
21.	Combination of verbal and sexual violence
22.	Combination of physical, verbal, and sexual violence (Triple type)
23.	No violence experienced

Note:

^a^this year = in the past 12 months

^b^in the past = more than 12 months prior

For the comparison and correlation tests, the attributes of violence selected were those whose values, when accumulated, did not cause circular computation. We performed correspondence analysis to describe subtle differences between the respondents’ characteristics by displaying them in a two-dimensional plot. We only used 11 attributes of violence, as we did for the bivariate analysis. We carried out the calculations with the help of generic spreadsheet software and SPSS v.26 [35].

## Results

Table 2 displays the respondents’ profiles based on several categories. Most of them lived in municipalities (56.2%), were at the middle school level (55.8%), were female (58.2%), were the eldest of their siblings (35.4%), lived with both biological parents (78.6%), and their mothers and fathers were high school graduates or higher (49.0 and 53.8%). In addition, most of the respondents identified as being of Sundanese origin (67.8%) and Muslim (94.1%).

**Table 2:** The respondents’ characteristics

Characteristics	Σ	%
City category		
City	1513	43.9
Municipality	1939	56.2
School-level		
Middle school	1926	55.8
High school	1526	44.2
Gender		
Male	1416	41.0
Female	2008	58.2
Did not answer	28	0.8
Child’s position in the family		
Eldest	1222	35.4
Middle	869	25.2
Youngest	1087	31.5
Only child	160	4.6
Did not answer	114	3.3
Number of siblings		
One sibling	1163	33.7
Two siblings	1091	31.6
More than two siblings	969	28.1
Did not answer	229	6.6
Home environment		
Both parents	2714	78.6
Father only	77	2.2
Mother only	266	7.7
Foster parents	26	0.8
Not a family home	369	10.7
Mother’s education		
Less than high school	1282	37.1
High school or higher	1692	49.0
Did not answer	478	13.8
Father’s education		
Less than high school	1035	30.0
High school or higher	1856	53.8
Did not answer	561	16.3

*N* = 3452

Table 3 describes the proportion of respondents according to their experiences of violence (*N* = 3452); 87.2% of them had encountered any type of violence in 2017 or prior. Of all respondents, 26% had experienced repeated violence in the past year as well previously, while 13.5% had experienced combined physical and verbal violence, physical and sexual violence, sexual and verbal violence, or all three types of violence. Physical violence in 2017, before 2017, or repeated violence was always more prevalent than verbal or sexual violence. More adolescents in middle school experienced violence than those in high school. Gender-based analysis indicates that violence was more prevalent among female versus male adolescents, except for verbal and sexual violence in 2017 before or after a repeated experience.

**Table 3:** Percentage of the respondents according to their experiences of violence

Violence	Total	City category		Level of school		Gender^**a**^	
		City	Municipality	Middle school	High school	Male	Female
Any violence this year	78.7	35.4	43.3	45.5	33.2	33.1	45.0
Any violence in the past	34.4	15.0	19.4	17.3	17.2	14.1	20.2
Any violence this year & in the past	26.0	11.7	14.3	13.4	12.5	10.9	14.9
Any violence, any year	87.2	38.8	48.4	49.3	37.9	36.3	50.3
Physical violence this year	78.2	35.1	43.1	45.3	32.9	32.9	44.7
Physical violence in the past	32.7	4.4	5.7	5.2	4.9	3.4	6.5
Physical violence this year & in the past	24.3	10.9	13.4	12.7	11.5	10.2	14.0
Verbal violence this year	12.2	4.4	5.2	5.5	4.1	5.0	4.5
Verbal violence in the past	5.9	1.9	3.0	2.3	2.6	2.3	2.5
Verbal violence this year & in the past	0.8	0.3	0.5	0.4	0.4	0.5	0.3
Sexual violence this year	4.5	1.5	1.8	2.3	1.0	2.0	1.3
Sexual violence in the past	2.7	0.7	0.8	0.6	0.9	0.8	0.7
Sexual violence this year & in the past	0.3	0.1	0.2	0.2	0.1	0.2	0.1

Note:

^a^0.8% of the respondents did not fill out the column for gender

The types of violence experienced by the respondents, timeframes, and offenders demonstrated that a small proportion of respondents did not state the type of violence (< 3.19%; see Supplementary Table 1). More female adolescents experienced being pinched to cause pain as the most frequent form of physical violence. In addition, more female adolescents experienced being spanked on the bottom with bare hands, given alcohol or drugs, kicked, and choked to prevent breathing. However, comparison analysis demonstrated no significant difference between male and female adolescents (*p* > 0.05).

Sexual violence is the least experienced form of violence, especially being made to watch a sex video or sexual pictures. All respondents who experienced violence stated who the offender(s) was/were, except for sexual violence, for which a proportion of respondents did not answer. Some respondents experienced physical or verbal violence from a combination of offenders; 0.97% (one case) experienced being burned, scalded, or branded by a hot object, while 11.26% were pinched to cause pain.

The share of physical, verbal, and sexual violence committed by children/adolescents was higher than that committed by adults compared to almost all forms of physical violence, except for the ear being twisted, being put in time-out, a meal being withheld for punishment, and being forced to engage in sexual intercourse. In addition, there were more adult male offenders than females that committed the following: slapping on the face or back of the head; hitting on the head with knuckles; hitting elsewhere (not the head) with an object; hitting multiple times with objects or fists; choking; burning with a hot object/water; locking up or tying up to restrict movement; forcing to stand, squat, or kneel to cause pain; putting in time-out; giving alcohol or drugs; kicking; threatening to hurt or kill; and all types of sexual violence. Male child/adolescent offenders exhibited similar patterns to adult male offenders, except for face/head-slapping, hitting elsewhere with an object, forcing a painful position, putting in time-out, and giving alcohol/drugs.

The OR analysis revealed violent attributes that differed significantly based on the respondents’ characteristics. School level, gender, living only with one’s biological mother, and parents’ education levels were significant differentiators for many violence attributes. During the year of the study, middle school students had a 1.8 times greater chance of experiencing sexual violence than high school students. Table 4 indicates that male respondents had a 1.993- and 1.553-fold greater chance of experiencing violence than females, including sexual violence in the study year and prior. Male respondents had a 1.688-fold higher chance of experiencing both physical and sexual violence, while they had a 2.738-fold greater chance of experiencing all three types of violence (physical, verbal, and sexual). Male adolescents had a lower chance of experiencing only one type of physical violence (0.593 times). Respondents who lived only with their biological father had a 2.255- and 2.073-fold higher chance of experiencing verbal violence or a combination of physical and verbal violence, respectively. Furthermore, those who lived only with their mothers had a 3.319 times greater chance of experiencing verbal violence in the study year and in the past. Gender was also a critical differentiator that determined the probability of various types of violence occurring.

**Table 4:** Odds ratios between respondents’ characteristics and violence attributes (only significant ORs [0 < 0.05] are shown)

Characteristic	City category^**a**^	Level of School^**b**^	Gender^**c**^	Respondent’s position in the family^**d**^				Number of siblings^**d**^	Home environment		Parents’ education levels	
											Mother^**e**^	Father^**f**^
Violence attribute	City	Middle School	Male	Eldest	Middle	Youngest	Only child	>Two	Father only	Mother only	< High school	< High school
At least one type, this year	1.235	1.467	1.186							1.521		
At least one type, past year		0.705		1.282			0.640			1.315	0.835	0.711
Any type, this year and past year		0.804		1.231						1.643	0.791	0.701
At least one physical form, this year	1.200	1.473	1.177							1.573		
At least one physical form, past year		0.733		1.404	1.215	1.243					0.853	0.691
Physical type, this year and past year		0.839		1.441						1.359	0.807	0.685
At least one verbal form, this year	1.252		1.806						2.255			0.732
At least one verbal form, past year		0.733										
Verbal type, this year and past year										3.319		
At least one sexual form, this year		1.811	1.993									
At least one sexual form, past year		0.660	1.553							2.153		
One physical form only		0.765	0.593		1.430		8.344					
More than one physical form		1.171									0.862	0.840
Physical and verbal type			1.388						2.073			
Physical and sexual type			1.688							2.217		
Verbal and sexual type												0.577
Triple type			2.738					1.797				
No violence experienced	0.752		0.740									

Notes: The numbers shown in this table are odds ratios that have *p* < 0.05 only

^a^Municipality OR = 1

^b^Highschool OR = 1

^c^Female OR = 1

^d^OR of each resulted from comparisons to other characteristics within the group

^e^High school or higher OR = 1

^f^High school or higher OR = 1

Since the outcomes of the comparison tests between each respondent characteristic with violence attributes demonstrated no difference, correlation tests (by calculating Cramer’s value) did not reveal a significant correlation; we performed correspondence analysis to describe subtle differences between groups.

**Fig. 1:**
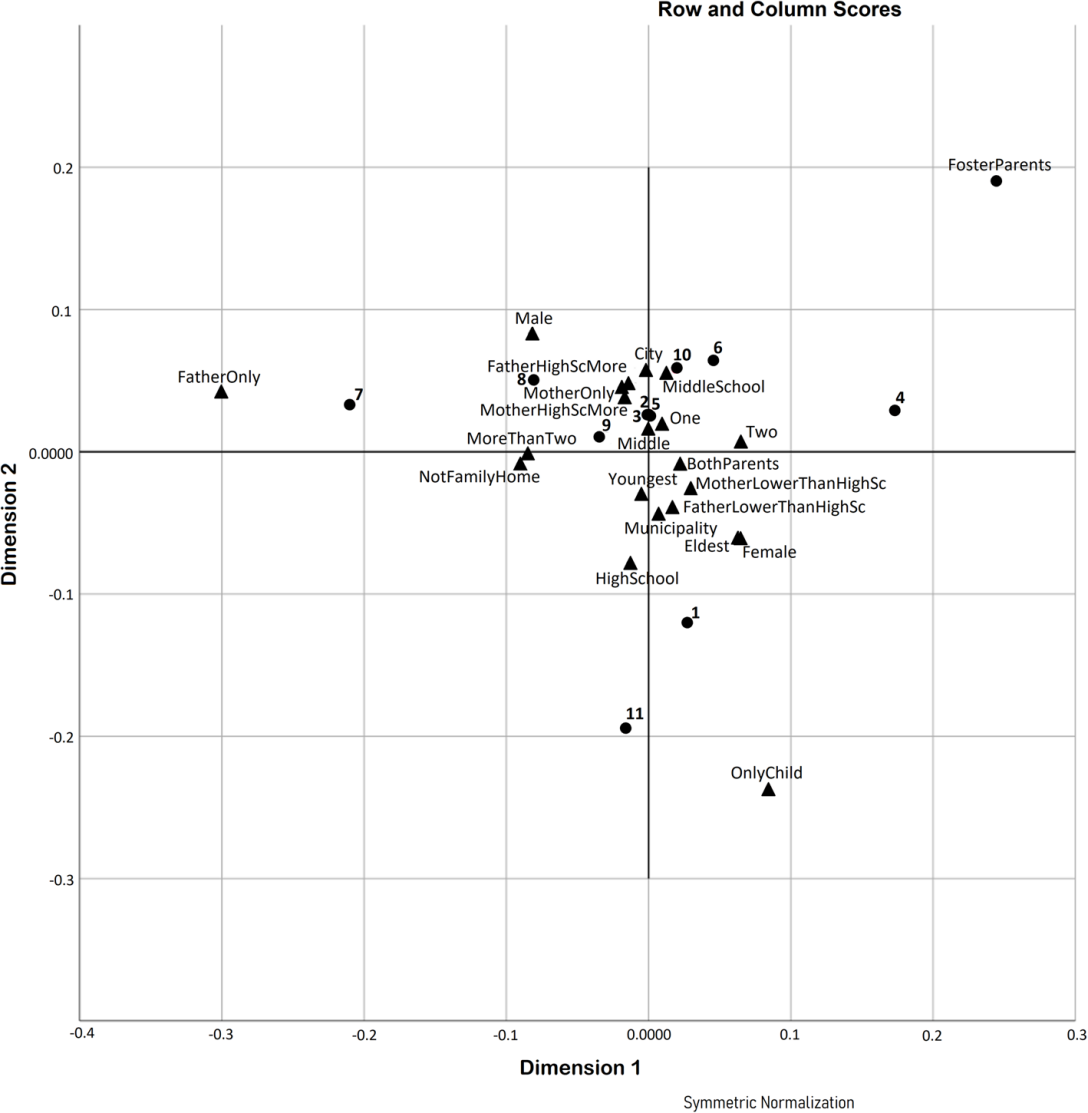
Correspondence analysis: 2D plot. This figure shows the correspondence analysis plot based on 11 violence attributes and respondent characteristics in the contingency table. Triangles refer to respondent characteristics, while points denote violence attributes. Number for each representation: (1) one type of physical violence only; (2) one type of verbal violence only; (3) one sexual type only; (4) physical and verbal violence only; (5) physical and sexual types only; (6) verbal and sexual types only; (7) physical and verbal violence only; (8) physical and sexual violence only; (9) verbal and sexual violence only; (10) triple violence; and (11) no violence was experienced

The correspondence analysis demonstrated subtle differences between groups of characteristics (see Fig. 1). The total inertia value in all characteristic groups was low (<0.0036), but successfully described a high percentage of data rooted in two dimensions. The analysis involved parents’ education level, number of siblings, the respondent’s position in the family, and the home environment by 100, 99.3, 96.7, and 92.0%, respectively. Dimensions 1 and 2 separate the groups in the plot to indicate the contrast between these groups. Grounded in the respondent’s position in the family, there is a contrast between the group of eldest children with the group of only children. Based on the number of siblings, dimension 1 indicates the contrast between adolescents who had two siblings with those who had more than two siblings. Regarding the home environment, dimension 1 contrasts adolescents who lived only with foster parents with those who lived only with their father. Rooted in parents’ education level, dimension 1 contrasts those whose fathers had less than a high school education with those whose fathers graduated from high school or higher.

## Discussion

We generated data on violence experienced by adolescents, especially physical violence; the numbers were much higher than those reported to the Indonesian Child Protection Commission. The findings indicate a rather worrying situation, with a high percentage of violence experienced in 2017 (78.7%); 26% of respondents experienced repeated violence in 2017 and previously. If we look further, the form of violence experienced most often is being pinched to cause pain (60.37%), followed by the ear being twisted (44.87%), which more likely results in minor injuries without permanent damage. However, in third place are forms of violence like hair being pulled (40.87%), the head being hit with knuckles (34.36%), and being kicked (29.81%), which have the potential to cause more severe injuries, not to mention stigmatising effects [36].

Table 3 outlines indications of physical violence at a much higher rate than that reported by similar studies in Indonesia and several developing countries, although we should note the differences in data collection approaches [13, 37–40]. In addition, the share of respondents who experienced physical violence in municipalities was higher than that in cities. This is in line with research in Ethiopia, but different from studies in other sub-Saharan countries and in Poland, which demonstrated a greater likelihood of urban adolescents engaging in violence than those in rural areas [41–43]. The suggestion from a previous study [13] that the prevalence of physical violence declines with age among adolescents is also supported by our results; we found that respondents in middle school (45.3%) experienced more physical violence than those in high school (32.9%).

Studies generally illustrate that most adolescents who experience violence are female, which is consistent with age and gender being well-known risk factors in victimisation [44]. In 2014, a survey by the FRA found that one in three women in the European Union older than 15 had suffered from physical or sexual violence [45]. Figures from 24 high- and middle-income countries show a prevalence between 8 to 31% among girls and 3 to 17% among boys under 18 [2]. Although Table 3 implies that physical violence was more prevalent among female respondents, the comparison test showed no significant difference between male and female adolescent victims (*p* > 0.05). This outcome is different from a previous study that signalled a higher prevalence of physical violence among males, which suggests a greater social tolerance for physical violence among men [43]. We do not think this finding alone can prove that the cities and municipalities in this study have gender inequitable societies, since this would require further investigation [41].

The results denote that children/adolescents were the foremost offenders of several types of violence (supplementary Table 1). The large proportion of offenders among adolescents is in accord with a meta-analysis study that supports the increasing findings of aggressive behaviour in children [46]. However, the violence report rate is relatively low in many countries, particularly if it depends on police forensic reports [38, 47]. The Child Protection Law in Indonesia mandates the obligation to report cases of violence against children; therefore, health professionals (or anyone) should not have any doubt to report such cases to legal authorities [48]. However, another report from Indonesia demonstrates that the extent to which healthcare professionals actively explore indications of sexual violence in daily practice is low [49].

While the Indonesian Criminal Law (Law No. 8/1981) rules on violent acts in general, the Child Protection Law (Law No. 35/2014 *juncto* No. 2/2002) regulates child protection more decisively. The Child Protection Law stipulates that violence against children comprises not only physical and sexual violence, but also psychological and neglect. It allows for the removal of parents’ guardianship rights for the child’s best interest, although as a last resort. It is illegal to leave a child in need of help due to violence; this crime carries a maximum penalty of five years of imprisonment or a fine (Article #78). Nevertheless, law enforcement is another matter. It is possibly related to the ignorance of healthcare professionals in detecting or reporting violence, and also to the difference in perception between them and law enforcement agencies regarding violence against women and children. For example, in practice, police tend to disregard the healthcare perspective that victim management should be conducted using a multidisciplinary approach [49].

From the perspective of Indonesian criminal law, sexual acts against children fall into several categories. To have sexual intercourse with a child under age 15 is a crime (the age of consent is 15 or older). It is considered an ordinary offence only if the child is under 12 years old; otherwise, it is a complaint offence (Indonesian Criminal Code, Article #287), except if the act caused injury or death (Article #288). The act of telling a child under 15 to watch, show, or touch his/her own or the offender’s private parts, or asking the child to make a sexually explicit video/take a sexually explicit photo, is categorised as molestation. However, it is classified as a complaint offence, except if the offender is the child’s guardian or if the act caused injury or death (Article #290). If it is a trafficking case, the consequence is 30% higher. This suggests that the questionnaire could not uncover dark numbers for this type of violence.

Although there was no significant difference between offenders of physical violence, if viewed in more detail, it appears that offenders were mostly peers, with male adolescents being the perpetrators of severe physical violence. This indicates a relatively stronger relationship pattern between respondents and adults versus respondents and peers. However, there are indications of corporal punishment from parents/guardians as a common approach to correcting children’s behaviour, as found by previous studies [37, 47], especially by male adults. We do not go into detail on the relationship between the offender and the victim, although this suggests problems in adolescents’ and adults’ perceptions of violence. The information that can be gleaned from the questionnaire is relatively limited. To better understand the phenomenon, in-depth analyses are required regarding the offenders (their relationship to the respondents and the number of offenders). The questions should go further, and a specific study should be conducted.

Previous research has shown that an act of violence is not necessarily considered violent by adolescents, which may contribute to underreporting. Verbal violence is often not perceived as criminal violence [50, 51]. This study shows that verbal violence experienced in the form of threats reached 10%, while a study among Mexican youths in 2017 found that figure to be 6% [52]. To threaten or to frighten a person is against two laws: the Indonesian Criminal Code (Article #335) for verbal threats, and the Indonesian Electronic Transaction Law (Article #29) for threatening or frightening a person with electronic media. These legal articles, however, can be applied only as a complaint offence.

Noticing the results indicating that offenders were predominantly male and female adolescents, we suggest that this is related to what has been suggested by Yusuf et al. regarding senior peer bullying, which requires interlevel intercultural interaction in schools [42]. We cannot ignore the prevalence of the threat ‘to be hurt or killed’ by male adults; female adults did not dominate the two forms of verbal violence studied. This tends to be seen as the influence of paternalistic culture, with unequal power dynamics between father/male adult figures who play a dominant role in instilling discipline in children, while the mother/female adult figure tends to provide a safe environment [37, 41]. Female adults and adolescents tend to scare victims by warning them of violence from others, while male adults and adolescents tend to make direct physical threats.

Concerning the possibility of parents’ education level as a risk factor, the findings reveal an interesting phenomenon. Low education among mothers or fathers (less than high school) implies up to a 0.685 times lower chance for respondents to experience several types of violence (see Table 4). Miller and McCaw implied that the relationship between poverty and violence is not straightforward and needs to be understood in a broader sociodemographic context. Furthermore, other factors may play a role. A low level of education is often considered to have a relationship with poverty. Women’s low level of education often causes them to become victims of violence, which will lead to the tendency for children to accept violent behaviour against women [53].

Some attributes of violence distinguish the child’s position in the family. Eldest and middle children had a greater chance (1.231 to 1.441 times) of experiencing physical and verbal violence, while only children tended to have a lower chance (0.640 times). Only children had a higher chance of experiencing only one type of violence (8.344 times). The finding that indicates the eldest’s higher chance of experiencing violence seems inconsistent with a study in Australia, which showed that younger siblings often become victims of violence perpetrated by other (older) siblings. That study suggested that most domestic violence (66%) is committed by adolescents in front of younger children [11]. The child’s position in the family is an interesting characteristic for further sociopsychological studies.

The high prevalence of violence revealed in this study indicates the practicality of the modified questionnaire. Nevertheless, we could not identify the manner of violence, whether it could be categorised as abuse, maltreatment, or peer bullying. Therefore, we recommend that the questionnaire be developed further to uncover the specific nature of violence. It is also necessary to inquire about the children’s own knowledge of their rights and motivation to report any violence experienced. In examining the offenders, we also think it is crucial not to limit the variables only to gender and age, but to extend them to the relationship between the offender and the victim.

Our study demonstrates risk factors for adolescents experiencing various forms of violence, which are very important for healthcare providers to increase their alertness during routine health examinations. We underline high risks amongst those who enter the early phase of adolescence in middle school, have more than two siblings, or live only with their mothers. Male adolescents should not be ignored because they face approximately the same risks as female adolescents of experiencing violence, including sexual violence. Male adolescents have a much greater risk than females of experiencing all three forms of violence (physical, verbal, and sexual). On the other hand, some characteristics are candidates for protective factors, such as an only child being less likely to experience profound violence.

There are several issues related to policies on handling violence against children in Indonesia: First, the general policy of authorities at the national and regional levels, policies regarding coordination between these authorities and law enforcement, and guidelines for health professionals as first responders. The Ministry of Women’s Empowerment and Child Protection (MoWECP) issued the Minimum Service Standard for Integrated Services for Women and Children Victims of Violence in 2010. However, the extent to which the standard has been implemented in healthcare services is still a question, since it was not issued by the Ministry of Health (MoH) as their supervising body. In 2009, the MoH last released a Guideline for Hospital Management of Integrated Services for Victims of Violence Against Women and Children, with a focus on victim management. A similar guideline for primary health care was also published in 2009, describing early detection or prevention only in general. In addition, the perception between the MoH and MoWECP with law enforcement on how health professionals, social workers, and the police should coordinate in practice is also still a question [49]. Our experience as medical practitioners in handling child victims shows that the recommendation to build a legal case is challenging, especially with the implication of separating children from their parents.

We did not collect data on the impact of violence experienced in terms of the respondents’ physics and psychology. It is necessary to further investigate the knowledge and motivation to report violence experienced by adolescents. In studying the offenders, we think it is crucial not to limit the variables to gender and age, but to extend them to the relationship between the offender and the victim. A more decisive analysis could explore the possibility of child abuse, domestic violence, school/neighbourhood bullying, and offenders’ characteristics. The assumption underlying parenting modes and adolescents’ socialising cultures used in this study needs to be clarified through in-depth socio-anthropological research to ascertain how these factors influence the incidence and types of violence experienced by adolescents. The bias due to the respondents’ position as minors, who still need parental permission to participate in research, is also a challenge in studies such as ours. In addition, because we conducted a school-based survey, we missed adolescents who did not attend school. Even though K-12 education is compulsory in Indonesia, the school dropout rate is high; separate studies are required to evaluate this phenomenon. We conducted this research before the COVID-19 pandemic; therefore, we could not cover what happens when adolescents stay home more, followed by intermittent school attendance starting in mid-2021.

## Conclusions and policy implications

Violence experienced by adolescents and the associated risk factors need to be further studied, as reports are usually incomplete or fragmented. Nevertheless, this research is expected to provide decisive suggestions for health professionals and organisations to strengthen case handling and prevention. Nevertheless, both issue-specific and broader coverage research are still required, including the sociocultural-legal context of violence in adolescents in this rapidly changing society. The problem is complex, and the correct knowledge, attitudes and actions are needed to carry out early detection, forensic examinations, and to manage the findings. An in-depth understanding of risk factors, healthcare knowledge of risk factors, the ability to detect a case, adolescents’ knowledge, their courage to report, and thorough forensic examinations are essential in overcoming the dark number, solving individual cases, and formulating policies by responsible authorities.

The results of our study are vital for advocating for the renewal of policies and service guidelines, because our study underscores the scale of the problem in the community to complement routine passive case identification. We propose that the MoH, MoWECP, and law enforcement agencies harmonise and renew related policies and guidelines. The findings reveal risks and protective factors essential for early detection and prevention guidelines for health professionals and social workers in primary health facilities. With the promising development of family medicine and the primary care profession in the last five years, we suggest that the outcomes can support authorities in taking decisive policy actions for front-line professionals in detecting, managing, and preventing violence against adolescents.

## Supplementary Information

**Additional file 1: Supplementary Table 1.** Types of violence, timeframe, and offenders.

## Data Availability

The datasets used and/or analysed during the current study are available from the corresponding author on reasonable request. All relevant scanned documents, such as ethical clearance, information for prospective participants/their parents, and an example of the informed consent form, will be provided upon request.
