# Supplementary material for: Cross-sectional survey of underreported violence experienced by adolescents: a study from Indonesia
Source: BMC Public Health. 2022 Jan 8;22:50. doi: 10.1186/s12889-021-12427-8 (PMC8742168; doi:10.1186/s12889-021-12427-8)
Supplement: Supplementary file 1 — Additional file 1: Supplementary Table 1. Types of violence, timeframe, and offenders. [file 12889_2021_12427_MOESM1_ESM.docx]

Supplementary Table 1 Types of violence, timeframe, and offenders

| **Type of violence** | **Time frame** | **Σ** | **% *** | **Offender** | **Σ** | **%** | **Type of violence** | **Timeframe** | **Σ** | **% *** | **Offender** | **Σ** | **%** |
| --- | --- | --- | --- | --- | --- | --- | --- | --- | --- | --- | --- | --- | --- |
| **1** | **2** | **3** | **4** | **5** | **6** | **7** | **1** | **2** | **3** | **4** | **5** | **6** | **7** |
| Being slapped on the face or back of head (n^**^=1283) | This year | 981 | 28.42 | Adult male | 233 | 18.16 | Being pinched and experiencing pain (n^**^=2477) | This year | 2084 | 60.37 | Adult male | 130 | 5.25 |
|  | In the past | 302 | 8.75 | Adult female | 154 | 12.00 |  | In the past | 393 | 11.38 | Adult female | 663 | 26.77 |
|  | Never | 2088 | 60.49 | Male child | 432 | 33.67 |  | Never | 867 | 25.12 | Male child | 233 | 9.41 |
|  | No answer | 81 | 2.35 | Female child | 420 | 32.74 |  | No answer | 108 | 3.13 | Female child | 1451 | 58.58 |
|  |  |  |  | Combination^***^ | 44 | 3.43 |  |  |  |  | Combination^***^ | 279 | 11.26 |
| Being hit on the head with knuckles (n^**^=1168) | This year | 912 | 26.42 | Adult male | 185 | 15.84 | Being forced to stand, squat, or kneel to cause pain (n^**^=516) | This year | 397 | 11.50 | Adult male | 178 | 34.50 |
|  | In the past | 256 | 7.42 | Adult female | 106 | 9.08 |  | In the past | 119 | 3.45 | Adult female | 76 | 14.73 |
|  | Never | 2189 | 63.41 | Male child | 506 | 43.32 |  | Never | 2853 | 82.65 | Male child | 67 | 12.98 |
|  | No answer | 95 | 2.75 | Female child | 329 | 28.17 |  | No answer | 83 | 2.40 | Female child | 195 | 37.79 |
|  |  |  |  | Combination^***^ | 42 | 3.60 |  |  |  |  | Combination^***^ | 27 | 5.23 |
| Being spanked on the buttocks with bare hands (n^**^=1445) | This year | 1186 | 34.36 | Adult male | 175 | 12.11 | Being put in time-out (n^**^=913) | This year | 699 | 20.25 | Adult male | 274 | 30.01 |
|  | In the past | 259 | 7.50 | Adult female | 216 | 14.95 |  | In the past | 214 | 6.20 | Adult female | 193 | 21.14 |
|  | Never | 1905 | 55.19 | Male child | 473 | 32.73 |  | Never | 2456 | 71.15 | Male child | 144 | 15.77 |
|  | No answer | 102 | 2.95 | Female child | 528 | 36.54 |  | No answer | 83 | 2.40 | Female child | 302 | 33.08 |
|  |  |  |  | Combination^***^ | 53 | 3.67 |  |  |  |  | Combination^***^ | 49 | 5.37 |
| Being hit on the buttocks with an object (broom, stick, cane) (n^**^=845) | This year | 641 | 18.57 | Adult male | 160 | 18.93 | Having a meal withheld as punishment (n^**^=156) | This year | 116 | 3.36 | Adult male | 40 | 25.64 |
|  | In the past | 204 | 5.91 | Adult female | 197 | 23.31 |  | In the past | 40 | 1.16 | Adult female | 56 | 35.90 |
|  | Never | 2513 | 72.80 | Male child | 216 | 25.56 |  | Never | 3231 | 93.60 | Male child | 25 | 16.03 |
|  | No answer | 94 | 2.72 | Female child | 230 | 27.22 |  | No answer | 65 | 1.88 | Female child | 35 | 22.44 |
|  |  |  |  | Combination^***^ | 42 | 4.97 |  |  |  |  | Combination^***^ | 6 | 3.85 |
| Being hit elsewhere with an object (broom, stick, etc.) (n^**^=870) | This year | 658 | 19.06 | Adult male | 184 | 21.15 | Being given alcohol or drugs (n^**^=153) | This year | 110 | 3.19 | Adult male | 49 | 32.03 |
|  | In the past | 212 | 6.14 | Adult female | 179 | 20.57 |  | In the past | 43 | 1.25 | Adult female | 13 | 8.50 |
|  | Never | 2504 | 72.54 | Male child | 236 | 27.13 |  | Never | 3243 | 93.95 | Male child | 45 | 29.41 |
|  | No answer | 78 | 2.26 | Female child | 220 | 25.29 |  | No answer | 56 | 1.62 | Female child | 46 | 30.07 |
|  |  |  |  | Combination^***^ | 51 | 5.86 |  |  |  |  | Combination^***^ | 6 | 3.92 |
| Being hit multiple times with object or fist (n^**^=636) | This year | 474 | 13.73 | Adult male | 126 | 19.81 | Being kicked (n^**^=1310) | This year | 1029 | 29.81 | Adult male | 284 | 21.68 |
|  | In the past | 162 | 4.69 | Adult female | 77 | 12.11 |  | In the past | 281 | 8.14 | Adult female | 100 | 7.63 |
|  | Never | 2738 | 79.32 | Male child | 298 | 46.86 |  | Never | 2054 | 59.50 | Male child | 622 | 47.48 |
|  | No answer | 78 | 2.26 | Female child | 118 | 18.55 |  | No answer | 88 | 2.55 | Female child | 304 | 23.21 |
|  |  |  |  | Combination^***^ | 17 | 2.67 |  |  |  |  | Combination^***^ | 52 | 3.97 |

Supplementary Table 1 (continued)

| **1** | **2** | **3** | **4** | **5** | **6** | **7** | **1** | **2** | **3** | **4** | **5** | **6** | **7** |
| --- | --- | --- | --- | --- | --- | --- | --- | --- | --- | --- | --- | --- | --- |
| Being choked to prevent breathing (n^**^=289) | This year | 212 | 6.14 | Adult male | 62 | 21.45 | Threatened to invoke harmful people (n^**^=427) | This year | 281 | 8.14 | Adult male | 75 | 17.56 |
|  | In the past | 77 | 2.23 | Adult female | 17 | 5.88 |  | In the past | 146 | 4.23 | Adult female | 104 | 24.36 |
|  | Never | 3087 | 89.43 | Child male | 157 | 54.33 |  | Never | 2961 | 85.78 | Male child | 110 | 25.76 |
|  | No answer | 76 | 2.20 | Child female | 49 | 16.96 |  | No answer | 64 | 1.85 | Female child | 138 | 32.32 |
|  |  |  |  | Combination^***^ | 4 | 1.38 |  |  |  |  | Combination^***^ | 30 | 7.03 |
| Being burned or scalded or branded (n^**^=103) | This year | 65 | 1.88 | Adult male | 26 | 25.24 | Threatened to be hurt or killed (n^**^=316) | This year | 227 | 6.58 | Adult male | 75 | 23.73 |
|  | In the past | 38 | 1.10 | Adult female | 15 | 14.56 |  | In the past | 89 | 2.58 | Adult female | 46 | 14.56 |
|  | Never | 3293 | 95.39 | Child male | 42 | 40.78 |  | Never | 3074 | 89.05 | Male child | 139 | 43.99 |
|  | No answer | 56 | 1.62 | Child female | 19 | 18.45 |  | No answer | 62 | 1.80 | Female child | 56 | 17.72 |
|  |  |  |  | Combination^***^ | 1 | 0.97 |  |  |  |  | Combination^***^ | 5 | 1.58 |
| Being forced to eat hot food to cause pain (pepper, etc.) (n^**^=290) | This year | 209 | 6.05 | Adult male | 41 | 14.14 | Being forced to look at someone else’s groin, or being forced to show one’s own (n^**^=184) | This year | 109 | 3.16 | Adult male | 25 | 13.59 |
|  | In the past | 81 | 2.35 | Adult female | 48 | 16.55 |  | In the past | 75 | 2.17 | Adult female | 4 | 2.17 |
|  | Never | 3103 | 89.89 | Child male | 69 | 23.79 |  | Never | 3211 | 93.02 | Male child | 96 | 52.17 |
|  | No answer | 59 | 1.71 | Child female | 123 | 42.41 |  | No answer | 57 | 1.65 | Female child | 7 | 3.80 |
|  |  |  |  | Combination^***^ | 9 | 3.10 |  |  |  |  | Combination^***^ | 0 | 0.00 |
| Being locked up or tied up to restrict movement (n^**^=363) | This year | 266 | 7.71 | Adult male | 86 | 23.69 | Being forced to touch, or being touched, in the groin (n^**^=114) | This year | 63 | 1.83 | Adult male | 15 | 13.16 |
|  | In the past | 97 | 2.81 | Adult female | 53 | 14.60 |  | In the past | 51 | 1.48 | Adult female | 8 | 7.02 |
|  | Never | 3024 | 87.60 | Child male | 116 | 31.96 |  | Never | 3288 | 95.25 | Male child | 49 | 42.98 |
|  | No answer | 65 | 1.88 | Child female | 99 | 27.27 |  | No answer | 50 | 1.45 | Female child | 9 | 7.89 |
|  |  |  |  | Combination^***^ | 9 | 2.48 |  |  |  |  | Combination^***^ | 0 | 0.00 |
| Ear being twisted (n^**^=2014) | This year | 1549 | 44.87 | Adult male | 394 | 19.56 | Being made to watch a sex video or sexual pictures (n^**^=37) | This year | 12 | 0.35 | Adult male | 4 | 10.81 |
|  | In the past | 465 | 13.47 | Adult female | 860 | 42.70 |  | In the past | 25 | 0.72 | Adult female | 2 | 5.41 |
|  | Never | 1357 | 39.31 | Child male | 146 | 7.25 |  | Never | 3364 | 97.45 | Male child | 8 | 21.62 |
|  | No answer | 81 | 2.35 | Child female | 493 | 24.48 |  | No answer | 51 | 1.48 | Female child | 2 | 5.41 |
|  |  |  |  | Combination^***^ | 120 | 5.96 |  |  |  |  | Combination^***^ | 0 | 0.00 |
| Hair being pulled (n^**^=1729) | This year | 1411 | 40.87 | Adult male | 143 | 8.27 | Being forced to have sexual intercourse (n^**^=48) | This year | 20 | 0.58 | Adult male | 11 | 22.92 |
|  | In the past | 318 | 9.21 | Adult female | 258 | 14.92 |  | In the past | 28 | 0.81 | Adult female | 6 | 12.50 |
|  | Never | 1613 | 46.73 | Child male | 314 | 18.16 |  | Never | 3356 | 97.22 | Male child | 7 | 14.58 |
|  | No answer | 110 | 3.19 | Child female | 912 | 52.75 |  | No answer | 48 | 1.39 | Female child | 2 | 4.17 |
|  |  |  |  | Combination^***^ | 102 | 5.90 |  |  |  |  | Combination^***^ | 0 | 0.00 |

**Notes:** ^*^ The percentage is based on N=3,452 ^**^ The percentage is based on n_i_ (the sum of respondents who answered ‘This year’ and ‘In the past’) ^***^ Offenders were a combination of male and female adults and/or adults and children. Analysis of variance showed no significant difference of offenders nor of victim’s gender in physical violence (p>0.05)
